# Three Cases of Dropsy of the Amnion

**Published:** 1879-07

**Authors:** F. C. Robinson


### Notes from Private Practice.

### Article V.

*Three Cases of Dropsy of the Amnion.*

In the February number of the Journal and Examiner appears an article from the pen of Gusserow, of Strasburg, upon the origin of the amniotic fluid which he claims to be exclusively a fœtal product, secreted by the kidneys of the child during the latter months of pregnancy. He bases his belief upon the fact that hippuric acid has been found in this fluid and therefore it must be derived from the urine of the child, because this acid is never found outside of that excretion. It may be possible for urine to be discharged into the amniotic cavity during the latter months of pregnancy, but that it is so discharged and is the cause of its accumulation there, I very much question. The amnion is formed from the external layer of the blastodermic membrane and contains a sero-albuminous fluid which constantly increases to accommodate the growth and movements of the fœtus. The quantity of this fluid varies in different pregnancies and when it accumulates sufficiently to cause premature labor or death of the fœtus, it then constitutes a disease denominated dropsy of amnion.

It is practically of no importance to the active practitioner whether the amniotic fluid comes from the child or from the mother as all therapeutical treatment is only palliative or positively useless as in the case herein reported, two of which occurred several years ago.

In November 1871, I was called to see Mrs. Watson, a large, muscular and well developed woman, age 36, suffering from an abdominal distention which she informed me had increased very rapidly during the last month, causing frequent palpitations of the heart, laborious respiration with inability to long retain a recumbent posture. She had transient pains in the loins and back and much tenderness over the uterus which seemed to occupy the whole of the cavity of the abdomen. She was in the eighth month of her fifth pregnancy. Diagnosis, dropsy of the amnion.

The following day she was taken in labor which made slow progress until the membranes were ruptured when an enormous quantity of fluid was discharged, flooding the patient, bed and floor, the amount of which I had no means of ascertaining. In two hours she was delivered of a still-born child weighing six pounds, perfect in limb and body but with its cerebrum entirely wanting and between the superciliary ridge of the frontal and the occipital protuberance no osseous formation, this area only covered by a loose and flabby integument giving the child a hideous and unsightly appearance. It was very difficult to ascertain the presenting position of this monster which probably had died before labor commenced when the mother had chills, several fainting fits and complete cessation of motion in utero. Under the use of ergot the uterus contracted firmly, expelling a normal placenta with only slight hæmorrhage, the mother making a perfect recovery.

In March 1872, was called to attend Mrs. Sappa, small, well formed blonde, age 24, in labor at full term of second pregnancy where the same dropsical condition occurred as in the case above reported, though in a minor degree. After a short labor a monster was born similar to the above, no brain or cranial bones above the ears; this surface covered by a loose skin well supplied with auburn hair. The child was alive at birth but never respired. The mother made a good recovery and has since passed a natural confinement with perfect child.

The following case is deserving of note because it shows that from whatever source this fluid is derived, the circumstances under which it accumulates are various and cannot be prevented or relieved by any systemic medication.

October 1st, 1878, was called to see Mrs. S., aged forty, six months advanced in third pregnancy, and suffering from an abdominal distention which seemed limited only by her capacity to still further enlarge. Diagnosis, dropsy of the amnion, prognosis, premature labor, certain treatment diuretics and cathartics. On the 3rd, she was taken in labor and upon rupturing the membranes more than three gallons of fluid were discharged into a vessel placed to receive it, soon followed by three perfect, and well developed boys of near six months fœtal life. There was one placenta with three amniotic apartments, which probably communicated with each other or were ruptured at the same time. The mother had some fever subsequently, yet made a perfect recovery.

The above cases suggest the following queries :

If liquor amnii is fœtal urine what caused it to accumulate so rapidly in those cases of imperfect development unless such an abnormal condition of fœtal growth increases urinary secretion, a fact which seems inconsistent with the known physiological action of secreting organs ? Were these blighted embryos caused by pressure of fluid cutting off the blood supply to the encephalon ? or did their atrophy produce such an accumulation ? Cannot this form of disease be explained by the laws of endosmose or exosmose of fluids, through or upon the surface of serous membranes when subject to irritation or inflammation ? If not, can three six months boys make three gallons of urine while in utero ?

Let the savans answer.

F. C. ROBINSON, M.D.

